# *BRAF p.V600E* genetic testing based on ultrasound-guided fine-needle biopsy improves the malignancy rate in thyroid surgery: our single-center experience in the past 10 years

**DOI:** 10.1007/s00432-022-04235-3

**Published:** 2022-09-07

**Authors:** Lei Gong, Yan Liu, Xinghong Guo, Chuan Wang, Fei Yan, Jinbo Liu, Xinguo Hou, Li Chen, Kai Liang

**Affiliations:** 1 Department of Endocrinology, Qilu Hospital of Shandong University, Jinan, Shandong China; 2 Institute of Endocrine and Metabolic Diseases of Shandong University, Jinan, Shandong China; 3 Key Laboratory of Endocrine and Metabolic Diseases, Shandong Province Medicine & Health, Jinan, Shandong China; 4 Jinan Clinical Research Center for Endocrine and Metabolic Diseases, Jinan, Shandong China

**Keywords:** Thyroid cancer, Ultrasound-guided fine-needle aspiration biopsy, *BRAF p.V600E*, Surgery, Malignancy rate

## Abstract

**Purpose:**

Ultrasound-guided fine-needle aspiration biopsy (UG-FNAB) was implemented in Qilu Hospital of Shandong University in 2015 as a preoperative diagnostic method for thyroid surgery. *BRAF p.V600E* genetic testing was implemented in 2019. This study evaluated the impact of these two tests on the malignancy rate in patients undergoing thyroidectomy.

**Methods:**

A total of 19,496 patients were included in the study. We retrospectively collected data from patients undergoing thyroid surgery in the Hospital Information System (HIS) of Qilu Hospital of Shandong University from January 2012 to December 2021. Meanwhile, data of FNAB, UG-FNAB, and *BRAF p.V600E* genetic testing were collected. Differences in means among groups were analyzed via one-way ANOVA, and differences in frequencies were analyzed via Pearson’s chi-squared test.

**Results:**

In this study, the 10-year period was divided into three stages, with the implementation of UG-FNAB in 2015 and that of *BRAF p.V600E* genetic testing in 2019 as dividing lines. The malignancy rate in thyroid surgery increased significantly during these three stages (48.06% vs. 73.47% vs. 88.17%; *P* < 0.001). In the same period (May 2019 to December 2021), the malignancy rate in thyroid surgery was significantly different between the Non-FNAB, UG-FNAB, and UG-FNAB-BRAF groups (78.87% vs. 95.63% vs. 98.32%; *P* < 0.001).

**Conclusions:**

The successful implementation of UG-FNAB and *BRAF p.V600E* genetic testing improved the malignancy rate in thyroid surgery and reduced unnecessary diagnostic surgery for benign and marginal lesions. It can, therefore, provide a clinical reference for other hospitals.

**Supplementary Information:**

The online version contains supplementary material available at 10.1007/s00432-022-04235-3.

## Introduction

In recent decades, the incidence of thyroid cancer in several countries has markedly increased, resulting in thyroid cancer representing 3% of the global incidence of all cancers (Li et al. 2020; Miranda-Filho et al. 2021). A recent study only found a rapid increase in the incidence of the papillary thyroid cancer (PTC) subtype, with small increases or decreases in other subtypes (follicular, medullary, and anaplastic) (Miranda-Filho et al. 2021). PTC is the main type of thyroid cancer, accounting for about 90% (Davies and Hoang 2021). The development of thyroid cancer is very complex, and the prognosis for thyroid cancers of different pathological types is very different, which requires standardized clinical management and treatment. In patients with indeterminate thyroid nodules, the subtype can be determined by diagnostic surgery, only 23% of postoperative pathologic findings are thyroid cancers, while the remaining 77% of patients undergo unnecessary thyroid surgery (Ianni et al. 2020).

Ultrasound examination is one of the most accurate physical diagnostic methods to assess the risk of malignancy in patients with thyroid nodules before surgical treatment (Russ et al. 2017). When thyroid nodules are suspected to be malignant by ultrasound examination, UG-FNAB is suggested to identify the benign and malignant nodules and provide a basis for the subsequent clinical treatment plan according to the 2015 American Thyroid Association (ATA) guidelines (Haugen et al. 2016). However, some thyroid nodules cannot be characterized by cytological examination. As a new diagnostic method, genetic testing using molecular markers (such as BRAF, RAS, and RET) is recommended for the auxiliary diagnosis of patients with thyroid nodules when cytological results are uncertain (Alexander et al. 2012; Duick et al. 2012). *BRAF p.V600E* mutation is the most common gene mutation in PTCs. Its incidence varies significantly with geography. Some studies suggest that *BRAF p.V600E* is associated with 40% to 50% of PTCs in the United States but is identified in up to 80% of PTCs in Asia (Yip and Sosa 2016). Previous review has shown that “Despite a high specificity for thyroid cancer, *BRAF p.V600E* mutation has a low overall sensitivity and therefore has a limited diagnostic value as a single screening test (Jinih et al. 2017)”. However, another study has pointed out that “*BRAF p.V600E* mutation is potentially useful adjunct to thyroid nodules, especially indeterminate samples classified as Bethesda System for Reporting Thyroid Cytopathology (BSRTC) III (Du et al. 2021)”. UG-FNAB and *BRAF p.V600E* genetic testing have been used in Qilu Hospital, but their effect on the incidence of malignant tumors during surgery in the past 10 years is not clear.

This study aimed to evaluate the impact of UG-FNAB and *BRAF p.V600E* genetic testing on the malignancy rate in thyroid surgery in Qilu Hospital, Shandong University, China, in the past 10 years to evaluate whether these methods can improve the determination of benign and malignant thyroid nodules, reduce the risk of thyroid cancer-related death and recurrence, reduce the potential harm of overtreatment for patients, and provide appropriate treatment and monitoring for high-risk patients.

## Methods

### Study population

Current study was accepted by the ethics committee of Qilu Hospital of Shandong University (ethical approval number KYLL-2018(KS)-226). The study methods were performed according to the ethical committee accepted guidelines. We retrospectively collected data from patients undergoing thyroid surgery in the Hospital Information System (HIS) of Qilu Hospital of Shandong University from January 2012 to December 2021. Meanwhile, data of FNAB, UG-FNAB, and *BRAF p.V600E* genetic testing were collected. Qilu Hospital of Shandong University is the largest general hospital in Shandong Province, with 5100 beds, 3,273,900 outpatient and emergency visits, 205,600 discharged patients, and 102,100 operations in 2021. Qilu Hospital is leading in endocrinology, pathology, ultrasound medicine, and other fields in China.

HIS data include information such as age, gender, thyroid ultrasound results, FNAB and *BRAF p.V600E* genetic testing results of thyroid nodules, pathological diagnosis of thyroid lesions, and operation date. A total of 19,496 patients were included in this study, including 4247 patients from the pre-UG-FNAB period and 15,249 patients from the post-UG-FNAB period. Among these 15,249 patients, 8008 patients underwent *BRAF p.V600E* genetic testing. This study did not include human investigations or animal experiments, so ethical approval was not required.

### Fine-needle aspiration biopsy diagnosis

An experienced FNAB operator palpated the thyroid, selected the puncture site, and disinfected the skin with alcohol. The nodule was fixed with the left hand, and the needle was inserted with the right hand. When the tip entered the nodule, the pin was pulled back, and the tip was moved rapidly back and forth in the nodule several times under negative pressure. When the tissue fluid was seen from the back hole of the needle holder, the negative pressure was released and the needle was immediately removed. Pressure was applied to the puncture site for 10 min. The aspirated material was placed on a slide, smeared, stained with Wright's stain, and examined under a microscope.

### Ultrasound-guided fine-needle aspiration biopsy diagnosis

An experienced FNAB operator and an ultrasound physician performed UG-FNAB. The ultrasonic probe was wrapped in a disposable protective film and disinfected. The patient was placed in a supine position. After the neck was disinfected with alcohol, the puncture site and route were selected according to the location of thyroid nodules indicated by ultrasound. The needle was inserted into the center of the nodule at 45 degrees and pulled up and down 10–20 times. The needle was quickly pulled out, and the fluid extracted from the needle was injected onto the slide. The smear was immediately fixed with alcohol, stained with hematoxylin–eosin, and sent to the pathology department for examination. The procedure was repeated as required by the total sample evaluation (usually two to three times per nodule). After sampling, a sterile gauze was used to press the puncture site for 30 min and medical observation was performed.

### Real-time PCR detection

The puncture tissue was stored in a 1.5-mL centrifuge tube containing 200 μL lysate buffer and sent to the Department of Pathology for analysis . Real-time PCR analysis was conducted with the *BRAF p.V600E* Mutations Detection Kit (Amoy Diagnostics Co., Ltd., Xiamen, China). After the PCR procedure was completed, the threshold was adjusted to a reasonable position, and data were interpreted according to the instructions.

### Statistical analysis

Continuous variables were expressed as the mean ± SD. Categorical variables were described as number (proportion). Differences in means among groups were analyzed via one-way ANOVA, and differences in frequencies were analyzed via Pearson’s chi-squared test. Two-tailed *P* < 0.05 was considered statistically significant in Table 1. In Tables 2 and 3, we conducted one-tailed test for Pearson’s chi-squared test and implemented Bonferroni method to address type I error rate related to multiple testing. Because pair-wise tests among three groups were compared for three times, the *P* value < 0.0167(0.05 divided by 3) was considered statistically significant in Tables 2 and 3. All data were analyzed using SPSS 22.0 (SPSS Inc., Chicago, IL, USA).

**Table 1:** Baseline characteristics of thyroid surgery patients in Qilu Hospital since 2012 to 2021

Characteristic	2012	2013	2014	2015	2016	2017	2018	2019	2020	2021	*P* value
Patients, *n*	1262	1413	1572	1667	1748	1837	1989	2334	2239	3435	–
Age (years), (mean ± SD)	48.28 ± 12.52	47.26 ± 12.70	47.26 ± 12.01	46.45 ± 12.08	46.83 ± 12.63	46.16 ± 12.60	45.80 ± 11.86	45.75 ± 12.50	45.23 ± 12.07	44.61 ± 11.86	< 0.001
Sex, *n* (%)											0.974
Female	965 (76.47)	1074 (76.01)	1187 (75.51)	1273 (76.36)	1315 (75.23)	1383 (75.29)	1517 (76.27)	1764 (75.58)	1675 (74.81)	2590 (75.40)	
Male	297 (23.53)	339 (23.99)	385 (24.49)	394 (23.64)	433 (24.77)	454 (24.71)	472 (23.73)	570 (24.42)	564 (25.19)	845 (24.60)	
Thyroid surgery, *n* (%)											< 0.001
Malignancy	454 (35.97)	668 (47.28)	919 (58.46)	1080 (64.79)	1238 (70.82)	1406 (76.54)	1596 (80.24)	1989 (85.22)	1969 (87.94)	3103 (90.33)	
Benign	808 (64.03)	745 (52.72)	653 (41.54)	587 (35.21)	510 (29.18)	431 (23.46)	393 (19.76)	345 (14.78)	270 (12.06)	332 (9.67)

**Table 2:** Management of thyroid nodules in Qilu Hospital in different periods

Characteristic	Pre-UG-FNAB	Post-UG-FNAB	
		Pre-UG-FNAB-BRAF	Post-UG-FNAB-BRAF
Period, year	2012–2014	2015–2018	2019–2021
FNAB, *n*	2769	1453	202
UG-FNAB, *n*		10,470	13,967
UG-FNAB-BRAF, *n*			5469
Thyroid surgery, *n*	4247	7241	8008
Malignancy, *n* (%)	2041 (48.06)	5320 (73.47)^a^	7061 (88.17)^a,b^
Benign, *n* (%)	2206 (51.94)	1921 (26.53)^a^	947 (11.83)^a,b^

^a^vs Pre-UG-FNAB, *P* < 0.001; ^b^ vs Pre-UG-FNAB-BRAF, *P* < 0.001. FNAB, non-ultrasound-guided fine-needle aspiration biopsy; UG-FNAB, ultrasound-guided fine-needle aspiration biopsy; UG-FNAB-BRAF, ultrasound-guided fine-needle aspiration biopsy and *BRAF p.V600E* genetic testing were performed

**Table 3:** The malignancy rate of thyroid surgery patients in Qilu Hospital in different groups since May 2019 to December 2021

Characteristic	Non-FNAB	UG-FNAB	UG-FNAB-BRAF
Patients, *n*	2674	1946	1965
Thyroid surgery, *n* (%)			
Malignancy	2109 (78.87)	1861 (95.63)^a^	1932 (98.32)^a,b^
Benign	565 (21.13)	85 (4.37)	33 (1.68)

^a^vs Non-FNAB, *P* < 0.001; ^b^vs UG-FNAB, *P* < 0.001. Non-FNAB, no fine-needle aspiration biopsy was performed; UG-FNAB, ultrasound-guided fine-needle aspiration biopsy; UG-FNAB-BRAF, ultrasound-guided fine-needle aspiration biopsy and *BRAF p.V600E* genetic testing were performed

## Results

### Characteristics of study participants

Patient characteristics are shown in Table 1. A total of 19,496 patients with thyroid nodules who underwent surgical treatment were included. The age at surgery decreased yearly, from 48.28 ± 12.52 years in 2012 to 44.61 ± 11.86 years in 2021 (*P* < 0.001). There were no significant changes in the male/female ratio. In 2012, there were 965 females (76.47%) and 297 males (23.53%), and in 2021, there were 2590 females (75.40%) and 845 males (24.60%) (*P* = 0.974). The malignancy rate increased yearly, from 454 cases (35.97%) in 2012 to 3103 cases (90.33%) in 2021 (*P* < 0.001).

### Changes in the number of patients undergoing thyroid surgery by thyroid core needle biopsy and gene testing

**Fig. 1:**
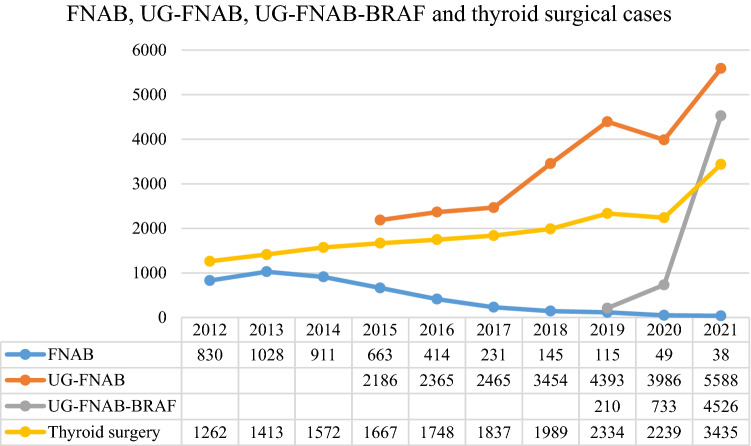
FNAB, UG-FNAB, UG-FNAB-BRAF and thyroid surgical cases in Qilu Hospital since 2012 to 2021. FNAB, non-ultrasound-guided fine-needle aspiration biopsy; UG-FNAB, ultrasound-guided fine-needle aspiration biopsy; UG-FNAB-BRAF, ultrasound-guided fine-needle aspiration biopsy and *BRAF p.V600E* genetic testing were performed

The changes in the numbers of thyroid surgery cases, FNAB cases, UG-FNAB cases, and *BRAF p.V600E* gene testing cases over the past 10 years are shown in Fig. 1. The number of surgical cases increased from 1262 in 2012 to 3435 in 2021. In 2020, due to COVID-19, there were fewer patient visits and significantly fewer surgeries. The number of surgical cases remained relatively stable or slightly increased. Qilu Hospital carried out UG-FNAB from 2015, and the number of UG-FNAB cases increased rapidly from 2186 cases in 2015 to 5588 cases in 2021. With the gradually increasing maturity of UG-FNAB technology, FNAB was basically replaced, and the number of FNAB cases decreased rapidly from 830 cases in 2012 to 38 cases in 2021. Qilu Hospital started *BRAF p.V600E* gene testing in 2019, and the number of cases increased rapidly from 210 cases in 2019 to 4526 cases in 2021.

### Malignancy rate in thyroid surgery

**Fig. 2:**
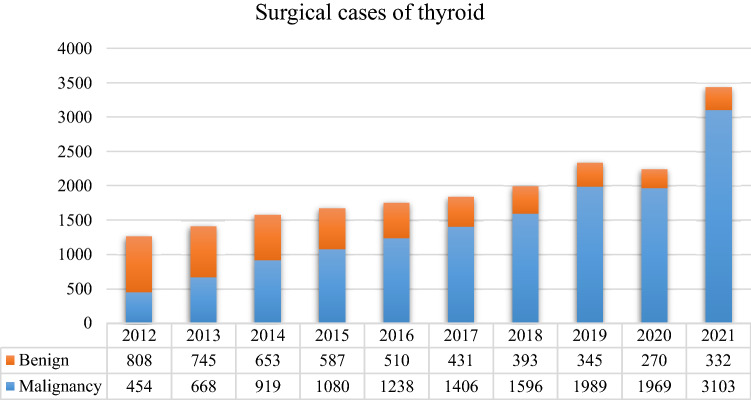
Benign and malignant surgical cases of thyroid in Qilu Hospital since 2012 to 2021

From 2012 to 2021, the malignancy rate in thyroid surgery increased yearly (Fig. 2). In 2012, before UG-FNAB was implemented, more than half (64.03%) of the patients treated surgically had benign tumors which did not require surgical treatment, and surgery was only performed for diagnostic purposes. UG-FNAB was implemented in Qilu Hospital in 2015, and the malignancy rate increased significantly. By 2018, only 19.76% of patients underwent surgery for benign lesions, especially non-neoplastic lesions, indicating that a large number of patients with benign lesions could be spared diagnostic surgery. Since 2019, when preoperative *BRAF p.V600E* gene testing was implemented in Qilu Hospital, the malignancy rate increased further. In 2021, only 9.67% of patients underwent surgery for benign lesions. In the last 10 years, we have gained more experience and achieved more effective surgical triage of patients with malignant tumors.

### Characteristics of patients undergoing thyroid surgery in different periods

The characteristics of patients in different periods are shown in Table 2. In this study, the 10-year period was divided into three stages, with the implementation of UG-FNAB in 2015 and the implementation of *BRAF p.V600E* genetic testing in 2019 as the dividing lines. Pre-UG-FNAB-BRAF stands for the period before *BRAF p.V600E* genetic testing was implemented. Post-UG-FNAB-BRAF stands for the period after *BRAF p.V600E* genetic testing was implemented. There was no difference in surgical procedures between the two periods. This study included 19,496 patients with thyroid nodules treated surgically: 4247 patients were treated in the pre-UG-FNAB period (2012–2014), and 15,249 patients were treated in the post-UG-FNAB period (2015–2021). Among these 15,249 patients, 7241 patients were treated in the pre-UG-FNAB-BRAF period (2015–2018), and 8008 patients were treated in the post-UG-FNAB-BRAF period (2019–2021).

We observed a significant difference in the malignancy rate between the pre-UG-FNAB and pre-UG-FNAB-BRAF groups (48.06% vs. 73.47%; *P* < 0.001) and between the pre-UG-FNAB and post-UG-FNAB-BRAF groups (48.06% vs. 88.17%; *P* < 0.001). We also observed a significant difference in the malignancy rate between the pre-UG-FNAB-BRAF group and the post-UG-FNAB-BRAF group (73.47% vs. 88.17%; *P* < 0.001).

### Characteristics of thyroid surgery patients in the same period

The characteristics of patients in the same period (from May 2019 to December 2021) are shown in Table 3. *BRAF p.V600E* genetic testing was implemented in Qilu Hospital in May 2019, so the data in this period were selected for analysis. Table 3 shows that the malignancy rate in thyroid surgery was significantly different between the Non-FNAB and UG-FNAB groups (78.87% vs. 95.63%; *P* < 0.001), between the Non-FNAB and UG-FNAB-BRAF groups (78.87% vs. 98.32%; *P* < 0.001), and between the UG-FNAB and UG-FNAB-BRAF groups (95.63% vs. 98.32%; *P* < 0.001).

## Discussion

Thyroid cancer is one of the most common endocrine malignant tumors. In 2020, 580,000 new cases of thyroid cancer were reported worldwide (Sung et al. 2021), making it the 11th most common cancer type. Thyroid cancer is expected to become the fourth most common cancer by around 2030. As shown in Table 1, during the 10-year period (2012–2021), the number of surgically removed malignant thyroid tumors soared from 454 to 3103, while the number of surgical beds increased only by 20 in Qilu Hospital. The latest data show that thyroid cancer incidence rates have increased in many countries and this epidemiological pattern has been largely attributed to an overdiagnosis effect (Pizzato et al. 2022). An increase in the number of thyroid cancers resected was observed in this study. However, this may or may not be the same as an increase in the incidence of thyroid cancer in the population. More accessible healthcare for example can account for more thyroid cancers coming to clinical attention without there actually being an increase in the incidence. The age of onset tended to decrease, with no significant change in gender ratio. Female patients accounted for about 3/4 of the total number of patients.

The prognosis of different types of thyroid neoplasms varies greatly (Cabanillas et al. 2016). Many large-sample studies have shown that clinical lymph node metastasis is an important factor affecting the survival of patients with thyroid cancer (Adam et al. 2015; Zhou et al. 2020). The prognosis of thyroid cancer is poor when the tumor has obvious extra-glandular invasion resulting in the invasion of surrounding important structures such as the larynx, trachea, and esophagus (Wang et al. 2016). Different treatment methods were selected according to different pathological types and clinical stages, such as active monitoring, affected side lobectomy plus isthmus resection, and total/near-total thyroidectomy (Filetti et al. 2019; Haugen et al. 2016). It is one of the most important challenges for clinicians to correctly diagnose benign and malignant thyroid nodules using effective diagnostic methods.

Current methods for differentiating benign and malignant thyroid tumors include ultrasound, preoperative FNAB, UG-FNAB, rapid intraoperative frozen section diagnosis, routine postoperative pathology, and molecular pathological examination (Filetti et al. 2019; Haugen et al. 2016). Ultrasound is the primary imaging method for risk assessment of thyroid nodules (Russ et al. 2017; Tessler et al. 2017). In the high-risk population (e.g., head and neck radiation exposure or radiation exposure in childhood, systemic radiation therapy) (Zupunski et al. 2019), thyroid ultrasound screening should be performed as early as possible. It has been suggested that the accuracy, sensitivity, specificity, positive predictive value, and negative predictive value of ultrasonography for malignant thyroid nodules were 68–70%, 84–91%, 33–49%, 67–71%, and 68–70%, respectively (Liu et al. 2021). The ATA guidelines indicate that the risk of malignancy for highly suspected malignant nodules is approximately 70–90%, and ultrasound results determine which nodules require fine-needle cytology biopsy (Haugen et al. 2016). FNAB is the first recommendation for suspected thyroid nodules due to its widely accepted safety and accuracy (Layfield et al. 2009).

Thyroid FNAB is gradually accepted in China. Some hospitals began applying UG-FNAB around the 1990s. At present, most general hospitals use thyroid UG-FNAB as a preoperative diagnostic method in China. Qilu Hospital is one of the thyroid FNAB microcosms among Chinese hospitals. Thyroid FNAB has been performed in the Department of Endocrinology in Qilu Hospital since 1991. UG-FNAB is performed for thyroid nodules since 2015. As shown in Fig. 1, with the implementation of UG-FNAB, the number of non-ultrasound-guided FNAB cases decreased sharply. However, the number of UG-FNAB cases increased rapidly from 2186 cases in 2015 to 5588 cases in 2021. It should be noted that these data are slightly affected by the impact of COVID-19 on medical treatment in China in 2020. But in 2021, COVID-19 had no discernible impact. Possible reasons for the increase in the number of thyroid UG-FNAB cases in the past 10 years are as follows: (1) Along with the rapid economic development, health awareness of people has improved, and health examination has become more popular in China. More asymptomatic thyroid nodules were detected. (2) Different national guidelines (Nabhan and Ringel 2017) and clinicians' personal experience lead to different nodular criteria for UG-FNAB selection. Guidelines for the implementation of FNAB have been published by the ATA and the National Comprehensive Cancer Network (NCCN) from the United States and the European Society for Medical Oncology (ESMO) and the European Thyroid Association (ETA) from Europe (Filetti et al. 2019; Haddad et al. 2018; Haugen et al. 2016; Russ et al. 2017). In all of these guidelines, FNAB is recommended for thyroid nodules sized ≥ 10 mm with a highly suspicious pattern. A recent study has indicated that the implementation of FNAB is considered necessary for a detailed and accurate assessment of the ultrasound appearance of thyroid nodules, rather than categorizing them solely on the basis of size (Shimura et al. 2021). (3) Patients are increasingly concerned about the nature of thyroid nodules and request UG-FNAB for thyroid nodules (Singh Ospina et al. 2020). As shown in Fig. 2, after thyroid FNAB was implemented in Qilu Hospital in 2015, the malignancy rate in thyroid surgery increased significantly (from 35.97% in 2012 to 64.79% in 2015). This suggests that preoperative UG-FNAB may reduce the number of unnecessary diagnostic thyroid surgeries. However, the inadequate or non-diagnostic aspiration rate of FNAB pathology are still relatively high (Chen et al. 2015).

*BRAF p.V600E* is the most common mutation in PTC. As *BRAF p.V600E* mutation leads to the continuous activation of the RAS–RAF–MEK–ERK pathway, it enhances the mitotic capacity of cells, ultimately leading to abnormal cell proliferation and tumorigenesis (Roth et al. 2018). Meta-analysis revealed that BRAF mutation is associated with lymph node metastasis, tumor stage, extrathyroidal extension, tumor size, and classic PTC (Li et al. 2012). In cases with a high *BRAF p.V600E* positive rate, such as patients diagnosed with PTC by FNAB, *BRAF p.V600E* genetic testing is unlikely to be helpful. In other cases, such as patients with uncertain and suspicious Bethesda classification, the presence of the *BRAF p.V600E* mutation may aid in diagnosis (Han et al. 2016). Molecular detection results need to be interpreted in combination with individual clinical manifestations, imaging results, and FNAB cytology results. After *BRAF p.V600E* genetic testing was implemented in Qilu Hospital in May 2019, the number of tests increased rapidly (Fig. 1). Meanwhile, as shown in Fig. 2, the malignancy rate in thyroid surgery increased significantly, from 85.22% in 2019 to 90.33% in 2021. These findings suggest that *BRAF p.V600E* testing may reduce the number of unnecessary diagnostic thyroid surgeries.

To further determine whether UG-FNAB and *BRAF p.V600E* genetic testing can improve the malignancy rate in thyroid surgery, we analyzed data on the malignancy rates in thyroid surgery during different time periods (Table 2) and within the same time period (Table 3). In this study, the 10-year period was divided into three stages, with the implementation of UG-FNAB in 2015 and the implementation of *BRAF p.V600E* genetic testing in 2019 as the dividing lines. There was a significant difference in the malignancy rate in thyroid surgery between the three stages (*P* < 0.001) (Table 2). Table 3 shows the data after the implementation of *BRAF p.V600E* genetic testing in Qilu Hospital. From May 2019 to December 2021, the malignancy rate in thyroid surgery was significantly different between the Non-FNAB, UG-FNAB, and UG-FNAB-BRAF groups (*P* < 0.001). These results suggest that the implementation of UG-FNAB and *BRAF p.V600E* genetic testing in Qilu Hospital has had a huge impact on the overall quality of life and healthcare costs of patients by reducing the number of unnecessary diagnostic thyroid surgeries. These unnecessary surgical excision of benign tumors mainly included nonfunctional, minimal thyroid adenoma, nodular goiter, thyroid cyst, etc.

There were likely other influences on the malignancy rate during this decade. For example, the gradual use of ultrasound evaluation system, Bethesda system for reporting thyroid cytopathology, optimization of cytological preparation method and FNA biopsy protocols, and more experienced and skilled operators may have influenced the diagnostic rate and ultimately malignancy rate in post-op specimens.

The current study has several limitations. First, this was a retrospective study, so the results may be biased. Second, our study is a single-center study, which cannot fully represent the overall situation in China but only describe a specific clinical experience. Third, this study only counted the number of thyroid operations, punctures, and *BRAF p.V600E* genetic testing cases and the malignancy rate in thyroid surgery in Qilu Hospital during the 10-year period, without further specific analyses of thyroid nodule ultrasound results, thyroid nodule size, metastasis, and other indicators. These will be considered in future studies. Fourth, our data showed that FNA and *BRAF p.V600E* genetic testing were selected according to doctors' experience, patients' wishes and medical expenses for thyroid nodule patients in the past 10 years, and these two examination methods may be overused.

In summary, the current study demonstrated that the implementation of UG-FNAB and *BRAF p.V600E* genetic testing in Qilu Hospital significantly improved the malignancy rate in thyroid surgery, reduced unnecessary surgical treatment, and optimized the initial treatment of thyroid cancer. Although this represents the clinical experience of a single center in China, it can provide a clinical reference for other hospitals.

## Supplementary Information

Below is the link to the electronic supplementary material.

Supplementary file1 (DOCX 42 KB)

## Data Availability

All authors declared that all data and materials as well as software application or custom code support their published claims and comply with field standards.
