# Supplementary material for: BRAF p.V600E genetic testing based on ultrasound-guided fine-needle biopsy improves the malignancy rate in thyroid surgery: our single-center experience in the past 10 years
Source: J Cancer Res Clin Oncol. 2022 Sep 7;149(8):4283–91. doi: 10.1007/s00432-022-04235-3 (PMC9450831; doi:10.1007/s00432-022-04235-3)
Supplement: Supplementary file 1 — Supplementary file1 (DOCX 42 KB) [file 432_2022_4235_MOESM1_ESM.docx]

**The method of BRAF gene analysis**

1. Composition of lysate buffer

10mM Tris-Cl (pH 8.0), 15mM NaCl, 10mM EDTA (pH8.0), 0.4% SDS.

2. Primer sequence and probe sequence for RT-PCR

| Primer | Sequence |
| --- | --- |
| BRAF-F-tag | tctgtagcAGCCCTCAGTAGCGAAGCAGTGATTTTGGTCTAGCTACAGA |
| BRAF-R-tag | AGCCCTCAGTAGCGAAGCAACTCAGCAGCATCTCAGG |
| T-primer | AGCCCTCAGTAGCGAAGCA |
| Exon-2-S10-tag | AGCCCTCAGTAGCGAAGCAGCACGAGTAACAAGCTCACG |
| Exon-2-R10-tag | AGCCCTCAGTAGCGAAGCAGATCATAATTCCTCTGCACATAGGTAA |
| E-2-S10 | GCACGAGTAACAAGCTCACG |
| E-2-R10 | GATCATAATTCCTCTGCACATAGGTAA |
|  |  |
| Probe | Sequence |
| BRAF-P-C | FAM-5’-TTCAAACCATCAGTTTGAACAGTTGTCTGGATCAACTG-3’- Tamra |
| Ex-2-P-C | 5-HEX-CTCTGGACAGCCTCCAGAGGATGTTCAATAACTGAACATC-3-BHQ2 |

3. Testing principle

Based on RT-PCR platform, this assay combines two technologies, specific primers and double-loop probes, to detect mutated genes contained in DNA samples. Specific primers were used to amplify mutant target sequences with high precision. Meanwhile, double-loop probes were used to detect the amplified products to realize the detection of mutations in sample DNA, so as to achieve high specificity and high sensitivity for rare mutation detection.

4. Method of determination

(1) Mix the reaction mixture in a vortex for 15 seconds, then centrifuge it quickly for 15 seconds. Mix the reaction mixture with the Taq enzyme in the proportion of 0.4μL Taq enzyme to 35μL per tube.

(2) Separate the reaction mixture with Taq enzyme into PCR reaction tubes by 35μL per tube.

(3) Add 5μL of DNA sample, positive quality control substance and negative control into PCR reaction tube successively, then carefully cover the PCR reaction tube. (DNA concentration: 0.4-1 ng/μL)

(4) Centrifuge PCR reaction tube to collect the reagents to the bottom of the reaction tube.

(5) Put the PCR reaction tube into the RT-PCR instrument.

(6) Open the settings window and set according to the amplification program diagram as shown in the following figure.


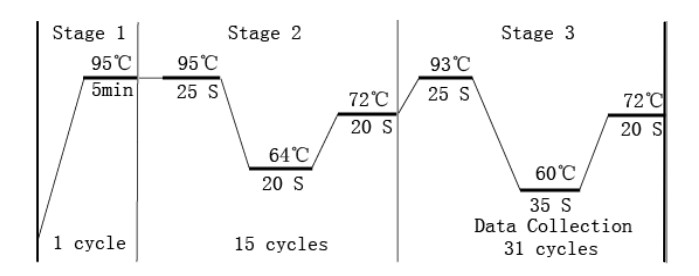


The first stage: 95 ^o^C for 5 min, one cycle;

The second stage: 95 ^o^C for 25S, 64 ^o^C for 20S, 72 ^o^C for 20S, 15 cycles;

The third stage: 93 ^o^C for 25S, 60 ^o^C for 35S, 72 ^o^C for 20S, 31 cycles;

Signal collection: In the third stage, FAM and HEX (or VIC) signals were collected at 60 ^o^C, RT-PCR was performed, and the files were saved.
